# Quality Improvement Project Investigating the Quality of Completed Section 5(2) Forms

**DOI:** 10.1192/bjo.2025.10355

**Published:** 2025-06-20

**Authors:** Ellen Cornish

**Affiliations:** Portsmouth Hospitals University NHS Trust, Portsmouth, United Kingdom

## Abstract

**Aims:** This quality improvement project aims to investigate the quality of completed Section 5(2) forms in a large, acute NHS hospital in England. It seeks to establish a current data baseline and identify common errors. The statutory section 5(2) form can be confusing for those who are unfamiliar with it, especially the section requiring correct deletion of options to identify the completing doctor’s status. Incorrectly completed Section 5(2) forms may later need rectification or can lead to the invalid detention of a patient, in which case the patient may be able to claim financial compensation.

**Methods:** The most recent twenty (n=20) Section 5(2) forms across adult and paediatric medicine from November to December 2024 were analysed against a created proforma containing twelve criteria needed to correctly complete the form and provide rationale for detention.

**Results:** On average Section 5(2) forms were 84% correctly completed with a total of 202/240 criteria met. Of the twenty forms surveyed, 100% were legally valid. Furthermore, 100% recorded diagnoses, symptoms, or behaviours suggestive of a mental health disorder and were legible, signed, and dated by the relevant parties. 70% identified risks to the patient or others if the patient were not detained and 55% contained correctly deleted phrases to reflect the status of Registered Medical Practitioner (RMP), Approved Clinician (AC) or Nominee. However, the majority (55%) contained medical abbreviations and only 40% indicated detention was necessary to allow a Mental Health Act Assessment (MHAA) to occur.

**Conclusion:** Overall Section 5(2) forms are completed well by doctors in this survey with all citing evidence of a mental health condition and the majority including an assessment of risk. Increased physician education and awareness of key information may increase the documentation of risks, the need for a MHAA and promote the avoidance of abbreviations which can cause errors. The ongoing work reviewing the new Mental Health Act could consider simplifying the pre-determined options, which may increase the correct completion of the RMP/AC/Nominee status section. Meanwhile, doctors may benefit from an aid with clear examples of the correctly deleted phrases being issued alongside the Section 5(2) forms. The surveyed hospital is currently revising Section 5(2) guidelines and preparing example templates for doctors to use. After allowing time for the implemented changes to take effect this project will aim to re-audit and measure impacts.

